# Reprogramming lipid metabolism in virus-infected neuronal cells

**DOI:** 10.21203/rs.3.rs-10472222/v1

**Published:** 2026-08-10

**Authors:** Luzhao Li, Haoran Qu, Wen Fu, Shu Feng

**Affiliations:** Cleveland Clinic; Cleveland Clinic; University of Southern California; Cleveland Clinic

**Keywords:** Viral infection, HSV-1, Zika, Neurodegeneration, Lipid metabolism

## Abstract

Exposure to environmental risk factors has been considered a major contributor to neurodegenerative diseases. As biological factors, infectious pathogens pose health concerns in the development of numerous aging-associated neurological defects. Emerging evidence continuously demonstrates the association between viral infection and the deterioration of brain function, such as dementia, multiple sclerosis, and microcephaly in infants. As intracellular pathogens, viruses demand macromolecules and energy by hijacking the host cell and reprogramming cellular metabolism. This study focuses on alterations in lipid metabolism in virus-infected neuronal cells using the DNA virus HSV-1 and the RNA virus ZIKV. We found that both HSV-1 and ZIKV downregulate lipid metabolism, sphingolipid metabolism, glycerophospholipid, and plasmalogen pathways in iPSC-derived neuronal cells, which may contribute to the pathogenesis of neurological diseases. The metabolomic dataset and analysis results will provide a valuable reference in studying the role of viral infection in neurodegeneration *in vitro* and *in vivo*.

## Introduction

Neurodegeneration imposes substantial health and socioeconomic burdens worldwide (1, 2). The pathogenesis is multifactorial, involving both genetic susceptibility and environmental influences. Numerous genetic mutations and chromosomal abnormalities have been directly linked to neurodevelopmental and neurodegenerative diseases (3, 4), whereas environmental factors, including microbial infection, toxic exposures, and lifestyle-associated insults, substantially influence brain homeostasis and disease development (5, 6). Among these environmental factors, microbial infection has emerged as an important contributor to neurological disease (7–9). For example, *Porphyromonas gingivalis*, the key pathogen of periodontitis, is strongly correlated with Alzheimer’s disease (10). It secretes virulent gingipains to penetrate the brain through the blood-brain barrier, trigeminal nerve, and gut-brain axis, triggering Aβ plaque deposition, tau tangles, and persistent neuroinflammation while provoking systemic inflammation and vascular injury to aggravate cognitive impairment (11). In addition, accumulating evidence indicates that neurotropic viral infections, including those caused by herpesviruses, flaviviruses, HIV, influenza A virus, and SARS-CoV-2, are closely associated with an increased risk of both neurodevelopmental and neurodegenerative disorders (12–15). Despite their distinct clinical manifestations, these viruses directly influence neural cells and induce persistent cellular stress responses that may contribute to neuropathology (16, 17). However, the mechanisms linking viral infection to long-term neural dysfunction remain poorly understood.

HSV-1 is a ubiquitous neurotropic double-stranded DNA virus, with more than 70% of the global population estimated to be seropositive (18). The virus is transmitted primarily through direct contact with infected oral or mucosal secretions and initially infects epithelial cells of the skin and mucosa (18). During primary infection, HSV-1 enters sensory nerve endings and is transported via retrograde axonal transport to the trigeminal or dorsal root ganglia, where it establishes lifelong latency (19). Periodic viral reactivation enables HSV-1 to spread back to peripheral tissues and to the central nervous system (CNS), particularly in the elderly, resulting in recurrent or asymptomatic brain infection and persistent neuroinflammation (20). Increasing clinical and experimental evidence suggests that persistent HSV-1 infection in the CNS is associated with amyloid-β deposition, tau hyperphosphorylation, and progressive neuronal dysfunction, implicating HSV-1 as a potential environmental risk factor for Alzheimer's disease (21–24).

Zika virus (ZIKV) is a positive-sense, single-stranded RNA virus belonging to the *Flavivirus* genus (25). As a mosquito-borne virus, ZIKV is transmitted primarily through the bite of infected *Aedes* mosquitoes and remains a significant global public health concern because of its widespread geographic distribution and potential for future outbreaks (26–28). Although most ZIKV infections are asymptomatic or cause only mild clinical manifestations, including fever, rash, headache, and malaise, infection during pregnancy can have devastating consequences (29). ZIKV readily crosses the placental barrier and infects the developing fetal brain, where it preferentially targets neural progenitor cells, resulting in impaired neurogenesis and congenital abnormalities, most notably microcephaly (30, 31).

As obligate intracellular pathogens, viruses cannot synthesize metabolites independently and therefore hijack host metabolic pathways including glucose, lipid, nucleotide and amino acid metabolism to acquire sufficient energy, synthetic precursors and membrane structural components for their own replication and assembly (32, 33). Among these pathways, lipid metabolism is particularly important for enveloped neurotropic viruses, which depend on host membrane remodeling throughout their life cycle (34, 35). Previous studies have shown that flaviviruses alter cellular lipid composition to facilitate replication complex formation, membrane biogenesis, and virion assembly (36–38). HSV-1 infection also disrupts host lipid homeostasis and membrane organization by altering phosphoinositide metabolism and phospholipid synthesis, processes that are important for viral envelope formation and efficient replication (39). However, the global landscape of lipid metabolic remodeling during HSV-1 infection in human neuro cells remains poorly characterized. In addition to supporting viral propagation, virus-induced metabolic alterations may also influence cellular stress responses, inflammatory signaling, and neuronal survival, raising the possibility that lipid metabolic remodeling contributes directly to neurological dysfunction in infected neural cells (40, 41).

Lipid homeostasis is essential for normal brain development and neuronal function. Neural cells require tightly regulated lipid metabolism to maintain membrane integrity, synaptic activity, myelination, and intracellular signaling (42–44). Increasing evidence indicates that disruption of lipid metabolic pathways contributes to both neurodevelopmental and neurodegenerative disorders. Altered fatty acids (45), glycerophospholipids (46, 47), sphingolipid (48, 49), and cholesterol homeostasis (50) have been associated with amyloid-β accumulation, tau pathology, neuroinflammation, and blood–brain barrier dysfunction in Alzheimer’s disease (51). Similarly, defects in sphingolipid biosynthesis can impair neural development and lead to severe congenital brain abnormalities, including microcephaly (52). These observations raise the possibility that virus-induced lipid metabolic dysregulation may represent a mechanistic link between neurotropic viral infection and neurological disease.

Human induced pluripotent stem cell (iPSC)-derived neural models provide a physiologically relevant platform for investigating virus–host interactions during human neural development (53). In this study, we use iPSC-based neural systems to examine how ZIKV and HSV-1 infection reshape host lipid metabolic pathways in human neural cells. We further explored the relationship between virus-associated lipid metabolic alterations and pathways linked to neurodevelopmental and neurodegenerative disorders. Our findings provide insight into how neurotropic viruses perturb lipid homeostasis in the human nervous system and may contribute to neurological pathogenesis.

## Methods and Materials

### Cell cultures

Vero cells, used for amplifying viruses, were purchased from ATCC and maintained in Dulbecco’s Modified Eagle Medium (DMEM) supplemented with 10% heat-inactivated fetal bovine serum (FBS) and 1% penicillin-streptomycin solution (Corning) at 37°C in a 5% CO_2_ humidified incubator. The neuron cells and neural progenitor cells (NPCs) were derived from the human iPSC line, from the fibroblast line AG14048 (Coriell) (54), and cultured in mTeSR medium (Stem cell technologies). To differentiate neurons, iPSCs were cultured onto a Matrigel-coated (Corning) plate with essential growth factors as previously reported with minor modifications (55, 56). In brief, iPSCs were initially infected with lentivirus encoding NGN2:2A:ASCL1 (Addgene Plasmid #127289), which encodes a doxycycline-inducible transcription factor cassette. To select cells, 0.5 μg/mL puromycin was added to the medium for 4 days. Selected iPSCs were further induced with neural induction medium (DMEM-F12, 0.5 × N2, 0.5 × B27 without VA, 1 × NEAA, 1 × GlutaMAX, 10 μM DAPT, 2 μM XAV939, 5 μM DMH1, 10 μM SB431542, and 2 μg/mL doxycycline) for 5 days. Lastly, these cells were passaged and seeded into Matrigel-coated 6-well plates. The cell number in each well is approximately 1 × 10^6^. Derived neurons were maintained in neuron maturation medium (DMEM-F12, 0.5 × N2, 0.5 × B27, 2 μg/mL doxycycline, 5 μM DAPT, 200 μg/mL cAMP, 10 ng/mL GDNF and 10 ng/mL BDNF) for 2 weeks before viral infection. To generate NPCs, iPSCs were singlized and seeded in mTeSR-E8 medium (Stem Cell Technologies) supplemented with 10 μM Rock inhibitor, at the cell density of 1 × 10^5^ per well. On day 1, medium was replaced with fresh E8 medium without Rock inhibitor in each well. After 2 days, cells were cultured in NIM-1A medium (DMEM/F12, 1×N2, 1×B27, 1×NEAA, 2 mM GlutaMAX, 10 ng/mL hLIF, 0.1 μM retinoic acid (RA), 4 μM CHIR99021, 3μM SB431542, 2 μM Dorsomorphin) for 5 days, and then in NIM-2A medium (DMEM/F12, 1×N2, 1×B27, 1×NEAA, 2 mM GlutaMAX, 10 ng/mL hLIF, 0.1 μM retinoic acid (RA), 4 μM CHIR99021, 3 μM SB431542) for another 5 days. The medium was changed every day. On day 9, cells were detached and grown in the maintenance medium (DMEM/F12, 1×N2, 1×B27, 1×NEAA, 1×Glutamax, 100 nM RA, 2 μM SB431542, 3 μM CHIR99021, 10 ng/mL bFGF and 10 ng/mL EGF) in non-adherent flasks for sphere formation. To passage cells, NPC spheres were collected by centrifugation at 100 × g for 1 min. Carefully remove the supernatant and add 5 mL Accutase to dissociate the cell pellet. After incubating for approximately 5 min, dissociate the spheres by carefully pipetting several times until the spheres were dissociated into single cells. In general, NPC spheres were passaged every 5–7 days. Seed cells into Geltrex-coated culture plate at a density of 1 × 10^6^ cells/mL for viral infection.

### Viruses

Human herpes simplex virus 1 (HSV-1, McKrea strain) was generously gifted by Dr. Konstantin G. Kousoulas. To amplify viruses, Vero cell monolayers were infected with HSV-1 stock at MOI = 0.01 for 48 hours as previously described (57). Virus-containing supernatant was harvested, filtered through a 0.45 μm cellulose filter, and pelleted via ultracentrifugation at 80,000 · g for 1 hour at 4°C. The pellet (HSV-1 virions) was resuspended in neuronal medium and titrated by plaque assays on Vero cell monolayers. Stocks were aliquoted and stored at − 80°C for further use. For infection, matured neurons were incubated with HSV-1-containing medium (MOI = 2) for 1 hour to synchronize the infection stage in cells. Medium was replaced with fresh neuronal medium, and cells were incubated for 16 hours before harvest for analysis.

Zika virus was purchased from ATCC (VR-84) and propagated in Vero cells. Monolayer Vero cells at 80% confluency were infected with Zika virus at MOI = 0.01 for 72 hours. Virus-containing supernatant was harvested and filtered through a 0.45 μm cellulose filter. Viruses were pelleted by ultracentrifuging at 150,000 · g for 2.5 hours at 4°C. The pellet was resuspended in 3E buffer (0.12 M tris-base, 0.06 M sodium acetate, 3 mM EDTA, pH 7.4) overnight at 4°C (58). Concentrated viruses will be titrated by plaque assays on Vero cell monolayers and diluted in maintenance medium for viral infection. For infection, NPCs were incubated with Zika-containing medium (MOI = 2) for 1 hour to synchronize the infection stage in cells. Medium was replaced with fresh maintenance medium, and cells were incubated for 8 hours and 16 hours before harvest for analysis.

### Metabolite analysis

Mock- and virus-infected cell samples were harvested and snap frozen in liquid nitrogen. Metabolite extractions were performed by the team at Metabolon, Inc.. Briefly, samples were prepared using the automated MicroLab STAR^®^ system (Hamilton Company). Several recovery standards were added to each sample prior to the extraction process for quality control. Methanol was added to each sample to precipitate protein and dissociate protein-binding small molecules, followed by vigorous vortexing for 2 min (Glen Mills GenoGrinder 2000) and high-speed centrifugation at 4°C. The supernatant was aliquoted for analyses by different Mass Spectrometry, including two separate reverse phase (RP)/UPLC-MS/MS methods with positive ion mode electrospray ionization (ESI), RP/UPLC-MS/MS with negative ion mode ESI, HILIC/UPLC-MS/MS with negative ion mode ESI, and backup. To remove the organic solvent, aliquot samples were placed briefly on a TurboVap^®^ (Zymark).

All extracted samples were reconstituted in solvents compatible to each method and analyzed with Ultrahigh Performance Liquid Chromatography-Tandem Mass Spectroscopy (UPLC-MS/MS, Waters ACQUITY ultra-performance liquid chromatography (UPLC) and a Thermo Scientific Q-Exactive high resolution/accurate mass spectrometer interfaced with a heated electrospray ionization (HESI-II) source and Orbitrap mass analyzer operated at 35,000 mass resolution).

Data extraction, compound identification, and analysis of metabolomics were performed by Metabolon. Normalized relative abundance of each metabolite is included in the supplementary material. Heatmaps were generated in R with the heatmap2 package. Pathway graphs were created with BioRender. Changes in specific lipid were created with Prism 11. Statistical analysis was performed using unpaired two-tailed Student’s t-test.: *P < 0.05; **P < 0.01; ***P < 0.001; ****P < 0.0001.

## Result

### Global lipidomic landscape alterations upon HSV-1 infection in human iPSC-derived neurons

To assess the impact of HSV-1 infection on lipid metabolism in cells, iPSC-derived mature neurons were infected with HSV-1 at an MOI of 2 for 16 hours and harvested for metabolite analysis. Z-score heatmap analysis revealed extensive alterations in lipid metabolism between mock and infected groups (Fig. 1A). Consistent with this observation, principal component analysis (PCA) showed clear separation of HSV-1-infected samples from mocks, indicating a substantial shift in the cellular lipid landscape upon infection (Fig. 1B). Volcano plot analysis identified widespread lipid alterations, with the majority of differential lipids showing significant reduction upon HSV-1 lytic replication (Fig. 1C). Pathway enrichment analysis further revealed that plasmalogen, sphingomyelin, glycerophospholipid (PC, PE, and PS), and fatty acid metabolism were among the most significantly affected pathways (Fig. 1D). These findings indicate that HSV-1 infection broadly remodels lipid metabolism in human neurons.

### Global lipidomic landscape alterations upon ZIKV infection in human iPSC-derived neural progenitor cells

To assess the impact of ZIKV infection on lipid metabolism in human neural progenitor cells, NPCs were infected with ZIKV at an MOI of 2 for 16 h and harvested for untargeted lipidomic analysis. Similar to HSV-1 infection, ZIKV induced widespread changes in lipid abundance, as demonstrated by Z-score heatmap analysis (Fig. 2A). PCA showed complete separation between infected and mock samples, highlighting the substantial impact of ZIKV infection on cellular lipid metabolism (Fig. 2B). Volcano plot analysis revealed that most differentially abundant lipids were decreased upon infection, whereas a smaller subset of metabolites was increased (Fig. 2C). Pathway enrichment analysis identified ceramide, sphingomyelin, plasmalogen, glycerophospholipid (PC, PI, and PS), and fatty acid metabolism as the most significantly altered lipid pathways (Fig. 2D). Together, these results demonstrate that ZIKV infection extensively remodels the NPCs lipidome.

### HSV-1 and ZIKV infections reprogram fatty acid metabolism in human iPSC-derived neural cells

Fatty acid metabolism profoundly regulates brain energy supply, neural development, and cognitive function. To determine how neurotropic viruses alter fatty acid metabolism in human neural cells, we analyzed metabolites involved in mitochondrial β-oxidation and polyunsaturated fatty acid (PUFA) pathways. Both HSV-1 and ZIKV infection (Fig. 3A) reduced the levels of free fatty acids and free carnitine (Fig. 3D) while increasing acylcarnitines and deoxycarnitine, indicating impaired carnitine shuttle activity and reduced mitochondrial β-oxidation. Accumulation of acylcarnitines in AD mice brain drives calcium overload, kinase-dependent tau hyperphosphorylation, mitochondrial dysfunction and neuroinflammatory responses (59). Defects in carnitine metabolism and β-oxidation have also been linked to impaired neural progenitor proliferation and abnormal brain development (60). These observations suggest that both DNA virus and RNA virus proceed through a similar path to reprogramming fatty acid synthesis leading to neurodegeneration.

In addition to β-oxidation defects, HSV-1 and ZIKV broadly downregulate PUFA metabolism. Multiple intermediates within both the ω−3 and ω−6 PUFA pathways were decreased during infection (Fig. 3B, 3C). Among these metabolites, eicosapentaenoic acid (EPA) (Fig. 3E), docosahexaenoic acid (DHA), and docosapentaenoic acid (DPA) levels showed a great reduction. In ZIKV-infected cells, DHA fell to approximately 16% of mock levels. DHA is highly enriched in neuronal membranes, and impaired DHA transport into the developing brain causes a severe microcephaly syndrome (61, 62). Similarly, reductions in DPA and EPA, two ω−3 PUFAs are observed, and it was also reported in neurodegenerative conditions (63). Together, these findings demonstrate that HSV-1 and ZIKV infection profoundly remodel fatty acid metabolism and disrupt lipid pathways important for normal neural development and function.

### HSV-1 and ZIKV infections disrupt sphingolipid metabolism in human iPSC-derived neural cells

Sphingolipid metabolism maintains brain cell membrane integrity, neuronal signal transmission and neuroinflammatory homeostasis (64). To investigate the effects of viral infection on sphingolipid metabolism, we profiled key intermediates in the ceramide synthesis pathway. Both HSV-1 and ZIKV infection induced accumulation of 3-ketosphinganine, accompanied by decreased levels of downstream dihydroceramide, ceramide, and sphingomyelin (Fig. 4A). This pattern is consistent with reduced flux through the de novo ceramide biosynthetic pathway. Sphingolipids are essential components of neural membranes and play important roles in neurogenesis, cell survival, and neuronal signaling. Deficiency of ceramide and sphingomyelin can impair neural development, causing defective primary cilium formation and congenital microcephaly (52, 65). The reduction of these metabolites upon ZIKV infection therefore represents a metabolic signature consistent with pathways implicated in congenital Zika syndrome. Altered ceramide homeostasis is also a recognized feature of neurodegenerative disease. Notably, increased ceramide levels have been consistently reported in AD patients and are associated with oxidative stress, mitochondrial dysfunction, and neuronal injury (66, 67). In contrast, HSV-1 infection in our model resulted in reduced ceramide abundance. This discrepancy may reflect differences between acute high-MOI viral infection and the chronic pathological processes that characterize neurodegenerative disease.

At 8 h post-infection, elevated ceramide and sphingomyelin production was observed in two ZIKV and HSV-1 samples, pointing to a dynamic pattern of initial upregulation followed by a reduction (Fig. 5B, C). Disrupted sphingolipid metabolism induced by HSV-1 and ZIKV infection suggests relevance to neurodevelopmental and neurodegenerative disorders.

### HSV-1 and ZIKV infections remodel glycerophospholipid and plasmalogen metabolism in human iPSC-derived neural cells

We next examined the effects of viral infection on glycerophospholipid and plasmalogen metabolism. Among all lipid classes analyzed, plasmalogens exhibited the most pronounced reduction upon HSV-1 infection and were also markedly decreased in ZIKV-infected cells (Fig. 5A–G). As major phospholipids in the brain, plasmalogens contribute to membrane stability, synaptic function, and protection against oxidative damage (68, 69). Reduced plasmalogen levels have been observed in brain tissues of AD patients, and plasmalogen deficiency directly elevates γ-secretase activity, ultimately boosting the production of Aβ peptides and thereby accelerating the progression of AD (70, 71). Although plasmalogen deficiency disrupts myelin architecture, impairs synaptic and intracellular signaling, and induces neuronal apoptosis, neuroinflammation in mice, its potential contribution to ZIKV-associated microcephaly remains unclear (72).

HSV-1 infection in neurons decreased the cellular phosphatidylcholine (PC), phosphatidylethanolamine (PE), phosphatidylinositol (PI), and phosphatidylserine (PS). These phospholipids are essential for membrane integrity, intracellular signaling, and synaptic activity, and their depletion resembles lipid abnormalities reported in the brains of AD patients (73–76). In ZIKV-infected cells, the decline of these lipids was also observed, although the changes were moderate. Reduced PC and PE levels have been linked with neurodevelopmental disorders characterized by epilepsy and microcephaly (77), whereas disruption of PI metabolism impairs radial glial cell integrity and cortical development (78). Interestingly, phosphatidylglycerol (PG) levels were upregulated by both viruses, although the functional significance of these changes in neurological diseases remains unclear. Overall, these results indicate that HSV-1 and ZIKV broadly remodel glycerophospholipid and plasmalogen metabolism in neural cells, affecting lipid pathways associated with both neurodevelopment and neurodegeneration.

## Discussion

Neurotropic viruses are capable of invading the central nervous system and causing a wide spectrum of neurological disorders. After initial infection of neural tissues, viruses can directly induce neuronal injury and cell death while simultaneously triggering immune cell activation and robust inflammatory responses that contribute to viral encephalitis and long-term neurological sequelae (79, 80). Beyond direct neuronal damage and inflammation, viral infection also reshapes host cellular metabolism in the brain, which serves as a critical driver of neuropathogenesis. Metabolic reprogramming is a central component of virus–host interactions in the nervous system, as neurotropic viruses actively remodel host metabolic pathways to satisfy the energy and biosynthetic demands of viral replication (81, 82). For example, rabies virus infection increases cerebral glucose uptake and accelerates tricarboxylic acid cycle activity, thereby promoting viral replication and exacerbating neuroinflammation and neuronal injury (83). ZIKV infection disrupts NAD^+^-dependent mitochondrial metabolism in the developing brain, leading to neuronal death and microcephaly (84), while virus-induced alterations in glycolysis and mitochondrial function can be partially rescued by pyruvate supplementation in infected pregnant animals (85). Similarly, HIV-1 infection perturbs cerebral glucose utilization and mitochondrial bioenergetics, resulting in chronic neuroinflammation and metabolic dysfunction associated with HIV-associated neurocognitive disorders (HAND)(82). These findings highlight the critical role of metabolic remodeling in the pathogenesis of virus-associated neurological disease. Compared with the well-established roles of glucose and mitochondrial metabolism in neurotropic viral infection, the contribution of lipid metabolic reprogramming to virus-associated neurological disease remains poorly understood, despite the essential roles of lipids in viral replication and neural homeostasis. In this study, we systematically characterized the alterations of lipid metabolites induced by HSV-1 and ZIKV infection in human iPSC-derived neural cells. Despite their distinct virological characteristics and clinical manifestations, both viruses disrupt the synthesis of fatty acids, sphingolipids, glycerophospholipids, and plasmalogens. Notably, many of the affected pathways contribute to the pathogenesis of neurodevelopmental and neurodegenerative diseases, suggesting that lipid metabolic dysregulation may represent a common host response to neurotropic viral infection and a potential mechanistic link between viral infection and neurological dysfunction.

Highly convergent lipidomic signature induced by HSV-1 and ZIKV was observed in this study despite their distinct evolutionary origins and replication strategies. HSV-1 (DNA virus) and ZIKV (RNA virus) belong to distinct viral families; both are enveloped viruses (86, 87), which may contribute to such lipid metabolic reprogramming. The replication of many enveloped viruses depends on extensive membrane remodeling, intracellular trafficking, replication organelle formation, and virion assembly and budding, all of which require substantial lipid resources from the host cell (88–90). In particular, sphingolipids and cholesterol are key components of membrane microdomains, commonly referred to as lipid rafts, which organize membrane proteins and signaling molecules and serve as important platforms for viral entry, assembly, and egress (91–93). Accumulating studies indicate that numerous enveloped viruses selectively exploit sphingolipid-rich membrane domains during their life cycle. HIV particles are enriched in sphingolipids relative to host plasma membranes, suggesting selective incorporation of lipid raft components during viral budding (94). Similarly, Flaviviruses bud inward into the endoplasmic reticulum lumen, so virions of flaviviruses such as West Nile virus contain envelopes highly enriched in sphingolipids (95). Among herpesviruses, mature HSV virions contain substantially higher levels of sphingomyelin than host nuclear membranes (96), while lipid rafts have also been implicated in the assembly of other herpesviruses, including HHV-6 (97). Furthermore, pharmacological inhibition of sphingolipid biosynthesis has been shown to impair HSV-1 replication, highlighting the functional importance of these lipids during infection (98). The central nervous system is particularly enriched in sphingolipids and other complex membrane lipids, which are essential for neuronal polarity, synaptic transmission, and membrane stability (99–101). Neurotropic enveloped viruses may preferentially exploit these abundant lipid resources to support replication within neural tissues. Consistent with their high demand for specialized lipids, both HSV-1 and ZIKV infection resulted in similar profound alterations in sphingolipid metabolism in our study. Nevertheless, lipid dependence is not a universal feature of all neurotropic enveloped viruses. For example, vesicular stomatitis virus and Semliki Forest virus appear to exhibit relatively limited lipid selection during envelope acquisition (102), suggesting that the extent of viral reliance on host lipid metabolism may vary according to viral replication strategy and assembly mechanisms.

Despite inducing broadly similar lipid metabolic alterations, HSV-1 and ZIKV are associated with markedly different neurological outcomes. One important explanation is the distinct biological contexts in which these viruses establish infection. ZIKV can cross the placental barrier and infect the developing fetal brain (103), where tightly regulated lipid metabolism is essential for neural progenitor proliferation, differentiation, and migration. Unlike ZIKV, HSV-1 rarely crosses the placental barrier and therefore has limited opportunity to directly disrupt fetal brain development during gestation (104). Instead, HSV-1 primarily infects mature neural tissues and establishes lifelong latency with periodic reactivation (16). Under these conditions, chronic disturbances in lipid metabolism may gradually impair neuronal function. Interestingly, both HSV-1 and ZIKV have been shown to replicate efficiently in human brain organoids and suppress organoid growth (105).

Several observations from our study differ from previous reports describing virus-induced lipid remodeling. Studies have reported elevated sphingolipid levels after ZIKV infection and demonstrated that sphingolipid metabolism is required for efficient viral replication (106). WNV infection has also been shown to increase levels of dihydroceramide, a key intermediate of de novo sphingolipid synthesis, and to enrich this lipid within viral particles, suggesting activation of host sphingolipid biosynthesis during infection(95). In herpesviruses, HSV-1 infection has been associated with enhanced ceramide synthesis and dependence on sphingomyelin metabolism (107, 108). plasmalogen also increased during HSV infection and this lipid accumulation supports efficient HSV-1 viral assembly and replication (109). In contrast, our lipidomic analyses revealed a broad reduction of sphingolipids, plasmalogens, and several other lipid classes upon both HSV-1 and ZIKV infection. These discrepancies may largely reflect differences in infection conditions. Our analyses were performed using a relatively high MOI infection model and samples were collected at 16 h post-infection, representing an acute phase of viral replication. Consistent with this interpretation, the sphingolipid precursor 3-ketosphinganine was elevated due to infection, and approximately half of the samples exhibited increased levels as early as 8 h post-infection (Fig. 4B, 4C). These observations suggest that de novo sphingolipid synthesis may initially be activated in response to viral infection. However, under conditions of rapid viral replication, membrane biogenesis, and virion assembly, newly synthesized lipids like sphingolipids and plasmalogens are likely consumed at a rate that exceeds their production, ultimately resulting in a net depletion of cellular sphingolipids.

Previous studies have demonstrated that pharmacological modulation of lipid pathways can effectively suppress viral replication. For example, ZIKV infection of primary human fetal astrocytes can be inhibited by GW4869, a specific inhibitor of neutral sphingomyelinase, highlighting the importance of sphingolipid metabolism for efficient viral propagation (110). However, most current studies focus primarily on restricting viral replication, whereas therapeutic approaches aimed at preventing or alleviating virus-induced neurological injury remain less explored. Our data revealed that both HSV-1 and ZIKV infection induced widespread depletion of multiple lipid classes, including sphingolipids, plasmalogens, and n-3 polyunsaturated fatty acids such as DHA and EPA. Because these lipids play essential roles in brain development, neuronal survival, and maintenance of neural function, restoration of lipid homeostasis may represent a complementary strategy for mitigating neurological damage after infection. Now, supplementation with exogenous DHA has been shown to rescue growth restriction and microcephaly in ZIKV-infected models (111). Similarly, plasmalogens have been reported to facilitate amyloid clearance and improve cognitive function in neurodegenerative and virus-associated neurological disorders (112). Thus, lipid metabolism may serve not only as a target for antiviral intervention but also as a potential therapy for preserving neural function and reducing long-term neurological sequelae after neurotropic viral infection.

There are several limitations in this study. First, our analyses were performed under acute infection conditions and therefore do not fully capture the long-term metabolic consequences of persistent or latent viral infection, particularly in the case of HSV-1 infection. Second, although human iPSC-derived neural cells provide a physiologically relevant model of human neural tissue, they cannot recapitulate the cellular and tissue complexity of the brain, including astrocytes, microglia, vascular cells, and infiltrating immune cells, all of which may substantially influence lipid metabolism during infection. Future studies should further investigate the mechanisms linking lipid dysregulation to virus-associated neurological disease by identifying the key metabolic enzymes controlled by the virus. In addition to their structural and metabolic functions, many lipids serve as important regulators of innate immunity, inflammatory signaling, and cellular stress responses. Therefore, virus-induced lipid remodeling may contribute to neurological pathology not only through disruption of neuronal homeostasis but also through modulation of neuroinflammatory responses within infected tissues. Combining genetic manipulation, lipid supplementation, brain organoid models, and in vivo infection systems will be important for determining whether restoration of lipid homeostasis can mitigate virus-induced neurological dysfunction. In addition to HSV-1, several herpesviruses, including varicella-zoster virus (VZV), Epstein–Barr virus (EBV), and HHV-6, have been associated with Alzheimer's disease and other neurodegenerative disorders (113–115). However, little is currently known about how these viruses remodel lipid metabolism in infected neural tissues. It would be important to determine whether the lipidomic signatures identified here extend to other members of the Herpesviridae family and contribute to their neurological manifestations.

## Supplementary Material

Supplementary Files

This is a list of supplementary files associated with this preprint. Click to download.

- SupplementaryTable.xlsx

## Figures and Tables

**Figure 1:**
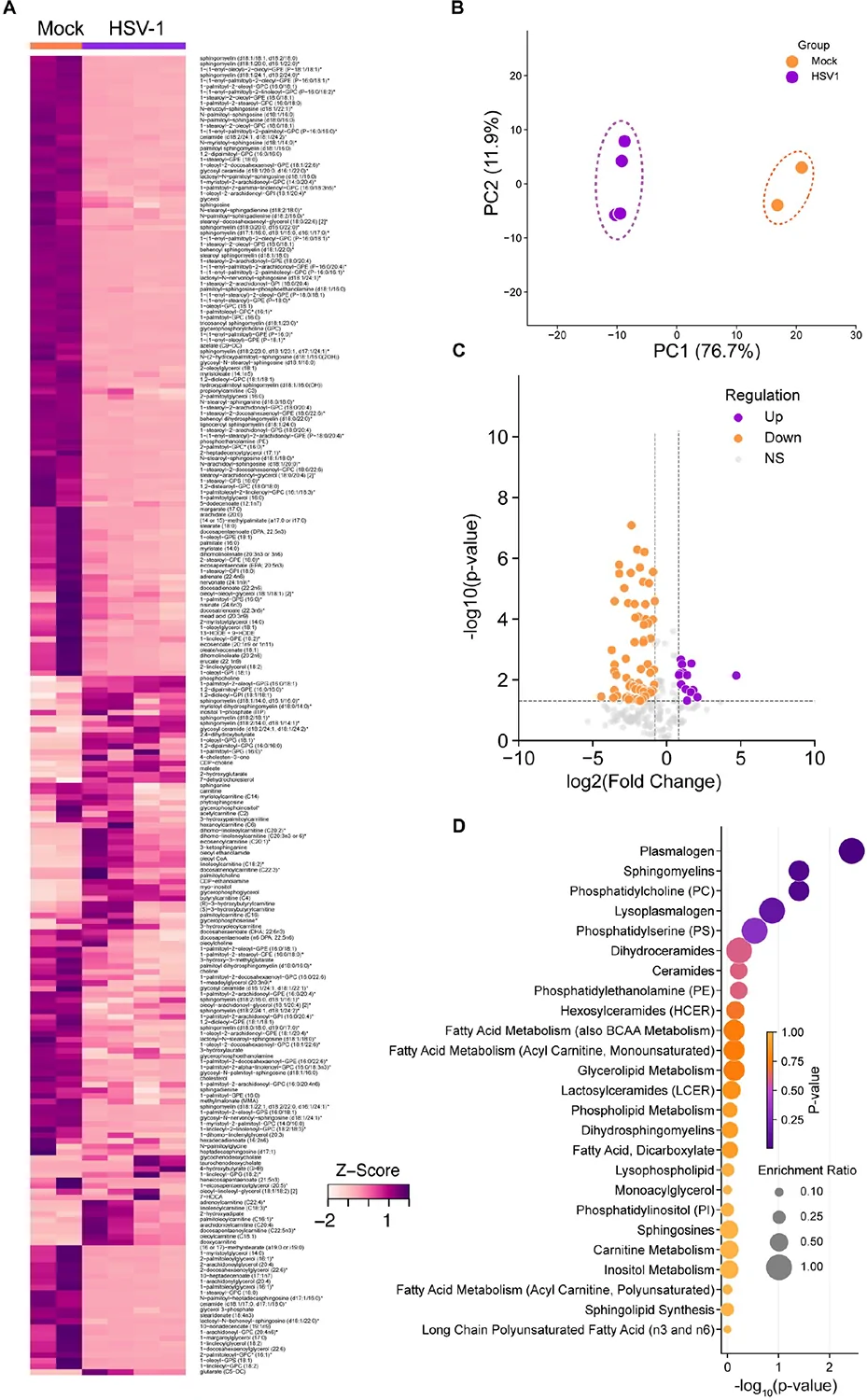
Global lipidomic alterations induced by HSV-1 infection in IPSC-derived neurons. (A) Heatmap of lipid metabolites in mock and HSV-1-infected cells. (B) Principal component analysis (PCA) of lipidomic profiles from mock and HSV-1-infected cells; purple, HSV-1; orange, mock. (C) Volcano plot of differential lipids showing log_2_ (fold change) versus −log_10_ (p-value). Each dot represents a lipid species; orange, significantly decreased lipids; purple, significantly increased lipids; gray, not significant (NS). (D) Pathway analysis of the top-enriched lipid metabolic pathways. Bubble size indicates enrichment ratio, and color represents pathway significance (p-value).

**Figure 2:**
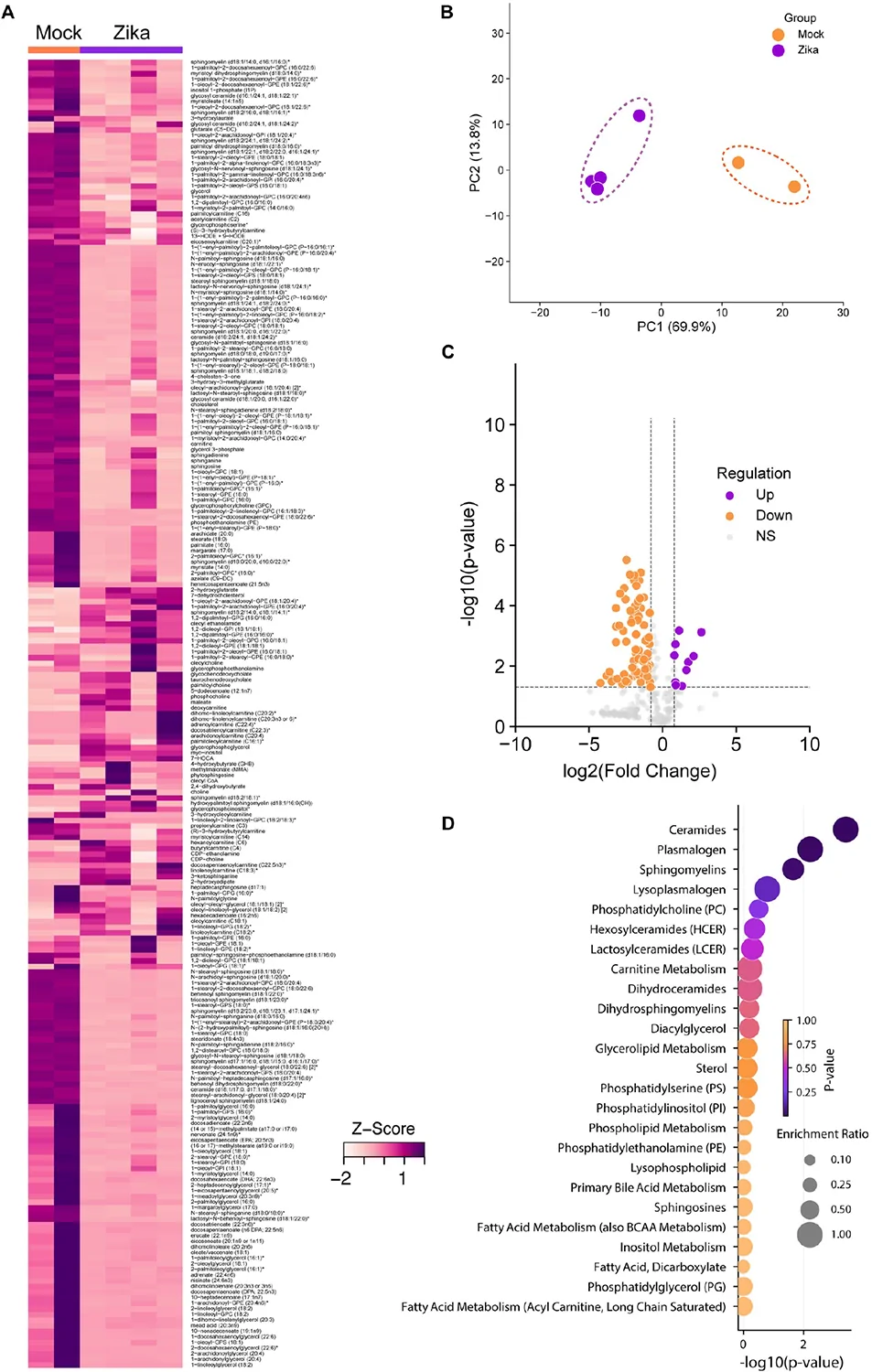
Global lipidomic alterations induced by ZIKV infection in IPSC-derived NPCs. (A) Heatmap of lipid abundances in mock and ZIKV-infected cells. (B) Principal component analysis (PCA) of lipidomic profiles from mock and ZIKV-infected cells; purple, ZIKV; orange, mock. (C) Volcano plot of differential lipids showing log_2_(fold change) versus −log_10_(p-value). Each dot represents a lipid species; orange, significantly decreased lipids; purple, significantly increased lipids; gray, not significant (NS). (D) Pathway analysis of the top-enriched lipid metabolic pathways. Bubble size indicates enrichment ratio, and color represents pathway significance (p-value).

**Figure 3:**
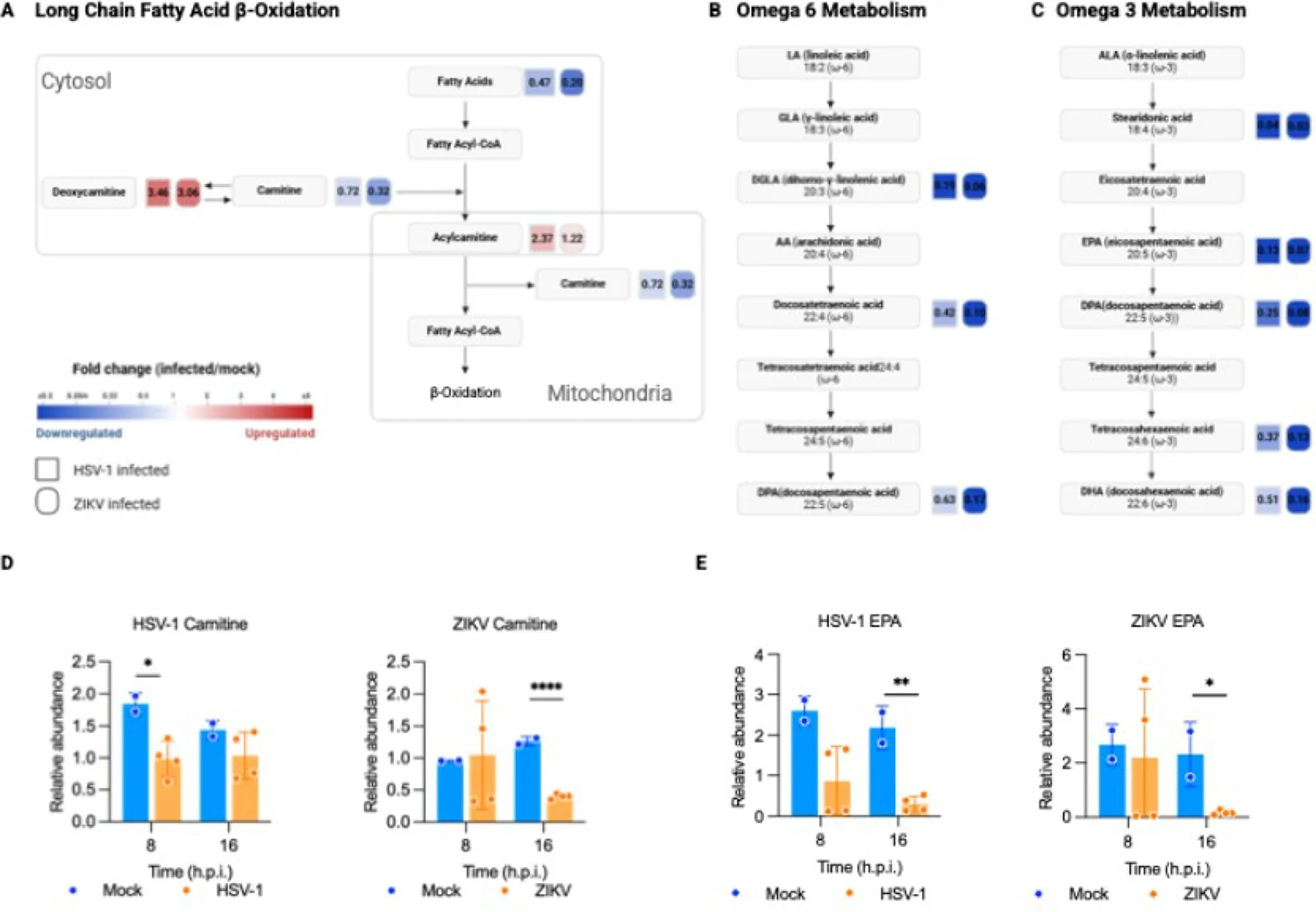
Both HSV-1 and ZIKV infections downregulate fatty acid metabolism in neuronal cells. (A) β-oxidation pathway showing fold changes (FC) in key metabolites upon HSV-1/ZIKV 16h infection. (B, C) n-6 and n-3 PUFA pathways with FC values the 16 h post-infection. FC: infected/mock; blue = downregulation, red = upregulation. (D, E) Changes in carnitine and EPA after HSV-1/ZIKV infection at 8 h and 16 h. Statistical analysis was performed using unpaired Student’s t-test.: *P < 0.05; **P < 0.01; ***P < 0.001; ****P < 0.0001.

**Figure 4:**
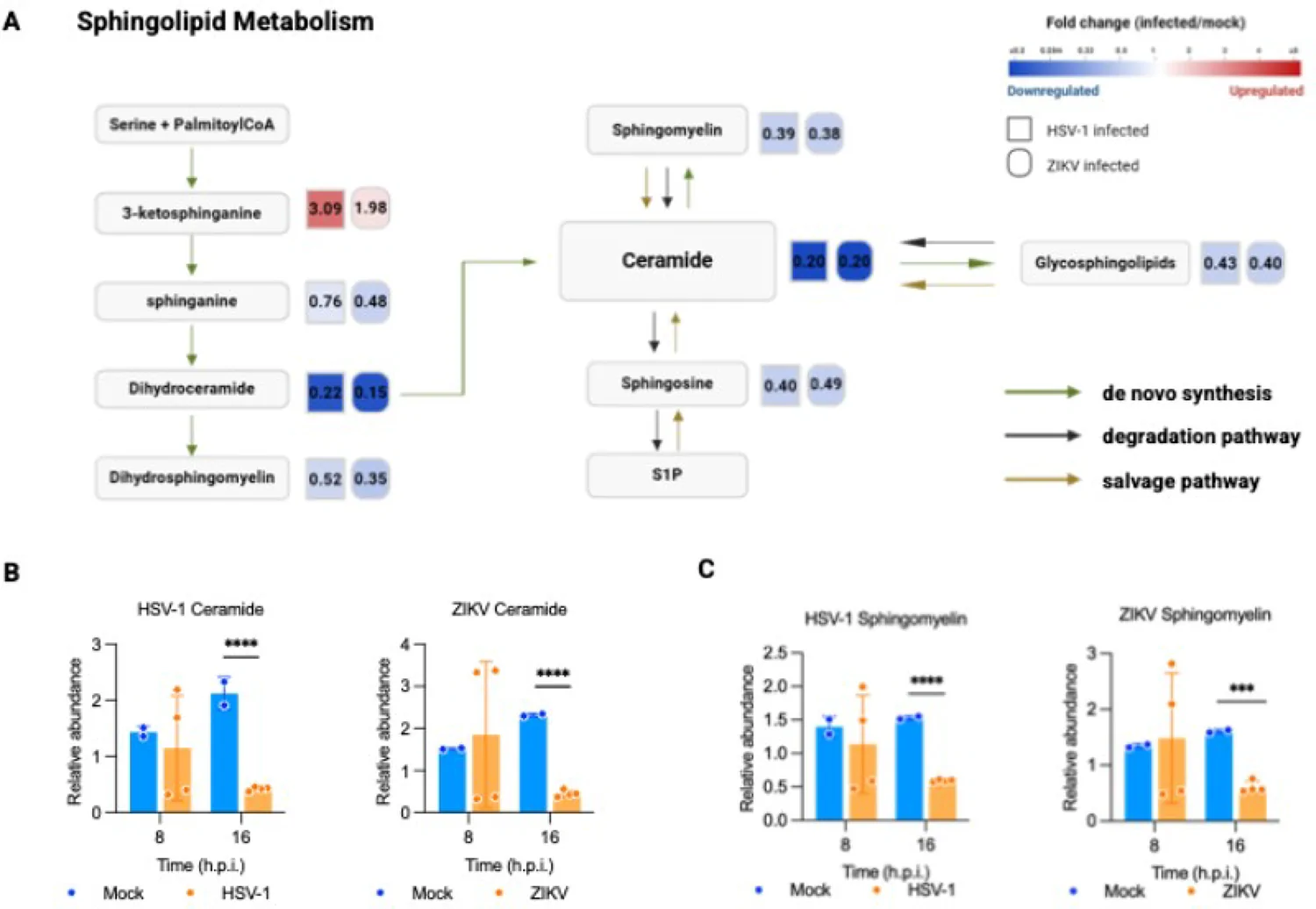
Sphingolipid-associated metabolites were significantly decreased in virus-infected neuronal cells. (A) Schematic overview of the sphingolipid metabolic pathway showing fold changes (FC) in key metabolites at 16 h post-infection. FC: infected/mock; blue = downregulation, red = upregulation. Arrows indicate de novo synthesis (green), degradation (black), and salvage pathway (gold). (B, C) Changes in ceramide and sphingomyelin after HSV-1/ZIKV infection at 8 h and 16 h. Statistical analysis was performed using unpaired Student’s t-test.: *P < 0.05; **P < 0.01; ***P < 0.001; ****P < 0.0001.

**Figure 5:**
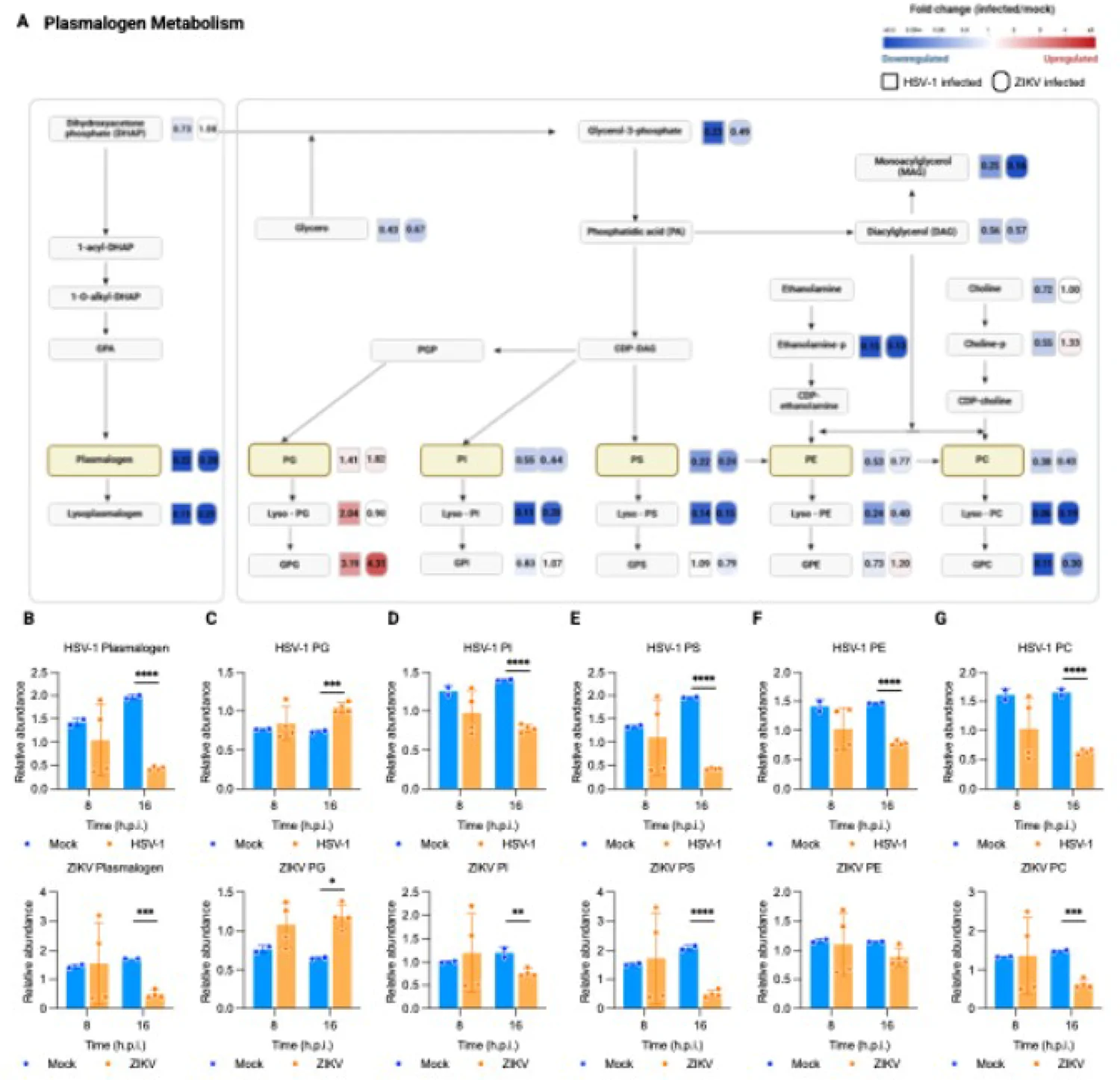
Viral infection reprograms glycerophospholipid and plasmalogen metabolism in human neuronal cells. (A) Schematic overview of glycerophospholipid and plasmalogen metabolic pathways showing fold changes (FC) in key lipids at 16 h post-infection. PC, phosphatidylcholine; PE, phosphatidylethanolamine; PS, phosphatidylserine; PI, phosphatidylinositol; PG, phosphatidylglycerol. FC: infected/mock; blue = downregulation, red = upregulation. (B - G) Changes in plasmalogen, PC, PI, PS, PE and PG after HSV-1/ZIKV infection at 8 h and 16 h. Statistical analysis was performed using unpaired Student’s t-test.: *P < 0.05; **P < 0.01; ***P < 0.001; ****P < 0.0001.

## Data Availability

The data that generated from this study are available within the article and its Supplementary Information files. Source data are provided with this paper.
